# An adolescent with significant emotional and medically unexplained complaints: case report and proposal of an intervention

**DOI:** 10.1186/s13034-015-0080-5

**Published:** 2015-10-15

**Authors:** Alemayehu Negash, Mubarek Abera, Christine Gruber-Frank, Reiner Frank

**Affiliations:** Department of Psychiatry, College of Health Sciences, Jimma University, Jimma, Ethiopia; Global Mental Health Group, Center for International Health, Ludwig-Maximilian University, Munich, Germany

**Keywords:** Child mental health, Ethiopia, Psychosocial intervention, WHO mental health GAP intervention guide, Collaborative international training programme, Conversion disorder

## Abstract

**Background:**

Ethiopia is a country in which child and adolescent mental health needs are often not met. In order to promote capacity building, a Collaborative International Exchange Programme has been established between Jimma University at Jimma, Ethiopia, and Ludwig-Maximilian University in Munich, Germany. The programme focuses on training non-physician health professionals in mental health speciality. One of the courses in the training programme, child psychiatry, involves a child psychiatrist and a children’s nurse supporting the management of a patient described in this case report. Its conceptual framework is based on the section “significant emotional and medically unexplained complaints” of the “WHO mental health GAP intervention guide for mental, neurological and substance use disorders in non-specialized health settings”.

**Objective:**

The purpose of this case report is to promote confidence in mental health professionals when managing patients with similar conditions, and to stimulate further evaluation of the conceptual approach in developing countries.

**Patient:**

The subject of this case report is a 14-year-old adolescent girl admitted to the psychiatric clinic at Jimma University Teaching Hospital. She was admitted for intractable retching, inability to eat, weight loss, and inability to walk. Challenges included the combination of medical and psychiatric symptoms, and the significant impairment of functioning in this adolescent. The first aim in the management of this patient was to guarantee vital functions. In a problem-oriented approach, different domains were addressed to restore nutritional, social, emotional, and motor functions. Treatment consisted of various elements of psychosocial interventions. The patient improved in 2 weeks and the final diagnosis was conversion disorder.

**Conclusion:**

Psychosocial interventions can be developed in cooperation, and applied in a setting where little child mental health expertise is available. Case-based learning relying on local expertise is suitable in meeting local needs and in developing mental health services for children and adolescents.

## Background

### Adolescent mental health

A review of articles on the epidemiology of mental disorders in children and adolescents found that approximately one in four young people had experienced a mental disorder during the preceding year worldwide and close to one-third across their lifetime [1]. Only 30 of 66 low and middle income countries were found to have a national policy incorporating children’s rights, and often these policies had a specific focus on abuse, rather than more general child mental health needs [2]. The regions in the world with the highest percentage of population under the age of 19 years are also those with the lowest level of resources [3]. Additionally, about half of all lifetime mental disorders begin before the age of 14 years [4].

Despite this, there is a paucity of trained professionals to meet the mental health needs of children and adolescents, and barriers to care which include poor identification of the problem, and lack of specialised personnel [2]. Inequality of access to such scarce resources is especially pronounced for children and adolescents with mental health problems, which reveals an unfortunate double disadvantage for mental health patients in low-income countries, specifically that the poorest countries spend the smallest proportion of their already scarce resources on mental health. Current evidence indicates that there are well-known barriers for young people with mental health issues in seeking proper handling and treatment. Rural populations, in particular, have severely inadequate access to mental health professionals in majority of low-income countries [5].

## Mental health in Ethiopia

In the Ethiopian context, mental health services are currently weakly organised and mental health capacity building is not satisfactory—though some efforts are being made. The available psychiatrists are disproportionately low in number and reside mainly in the capital city of the country. Furthermore, some psychiatrists have left the country to work abroad [6]. According to the National Mental Health Strategy of 2010, there were only 40 psychiatrists in Ethiopia. Of these, 10 were found to work in rural regions, and 30 in the capital, Addis Ababa. The rate per population is 0.04 % per 100,000 inhabitants [6]. In a country of more than 90 million inhabitants, there are only two child psychiatrists settled in Addis Ababa. Resources for non-medication alternatives, e.g. psychosocial rehabilitation and psychological treatment, are limited. Retention of qualified non-physician staff is also a problem due to the absence of a well-designed pre-set career development structure. Supervision, with defined output and ongoing training, is far below the minimum required. Furthermore, lack of recognition of child mental health problems by health professionals impairs the referral linkage from primary to specialist care [7]. Hope is offered by the recent expansion of non-physician mental health specialist training programmes at both the local and national level—even if they are still in their initial phases. Case studies can be of special use here in contributing to developing and evaluating specialised treatment protocols [3, 6, 8].

## Study setting

The Department of Psychiatry at Jimma University Teaching Hospital (JUTH) is guided by three psychiatrists, and additionally staffed by four masters in clinical and community mental health specialists, and nursing staff. There are 26 beds for inpatients, and the facility also provides an outpatient department. The clinicians have a good level of expertise in diagnosis and in treating patients with Psychoses, Depression, and Suicidality, as well as other disorders of adult psychiatry. The local language is Oromifa, though Amharic, the official language of the country is spoken widely. English is used as the clinical and scientific language. As a consequence in clinical practice, a series of translation processes takes place.

In a response to the low level of trained manpower, the Department of Psychiatry at Jimma University has been running a two-year Master of Science (MSc) Program in Integrated Clinical and Community Mental Health speciality since 2009, with the aim of building capacities in mental health. Candidates are not physicians, but have training as public health professionals (health officer) or nurses. It is a requirement that they complete full clinical service in the psychiatric hospital, supervised by the psychiatrists. In addition, there are 14 courses of 2 weeks duration, which cover different aspects in the field of psychiatry. The programme is supported by an exchange programme between the medical colleges of Ludwig-Maximilian University, Munich, Germany, and Jimma University [8, 9]. A detailed description of the course in child psychiatry is given separately [8], but it is worth mentioning that video recordings are used to train staff in recognition of nonverbal communication. This training is particularly relevant for child mental health problems. During the period of hospitalisation of the young patient, a professor of child and adolescent psychiatry and a senior children’s nurse were present at the psychiatric hospital as guest lecturers.

## Conceptual framework: WHO mental health GAP intervention guide

The WHO mental health GAP intervention guide for mental, neurological, and substance use disorders in non-specialised health settings (WHOmhGAP 2010) is a manual for priority conditions in mental health in developing countries, and it is used in the MSc program for training purposes. It was specifically used in the recognition and management of the patient discussed in this case report [10]. The WHO guide is intended for health professionals in general practice, but it is also useful for specialists in mental health. The introductory chapter, “General principles of care”, summarises key elements of good clinical practice, and therefore applies to all conditions. A good bedside manner and a respectful attitude towards patients and family constitute the basis of clinical skills. Only a few hours of training are necessary to be able to apply such “general principles of care”. “Advanced interventions”, as described in the last section of the WHOmhGAP, require longer training, and more staff time to implement. For each type of disorder, flowcharts describe what has to be assessed, how to make a decision, and how to manage the disorder specifically.

In children, treatment has to focus on psychosocial interventions, as medication is considered as not applicable by health professionals for general care in the WHOmhGAP intervention guide, and also in the Ethiopian National Mental Health Strategy [6, 10]. The WHO model list of essential medicines for children up to age 12 covers chlorpromazine and haloperidol for psychotic disorders, and fluoxetine for depression (2011) [11].

### Purpose of this paper

There is a paucity of publications on psychosocial interventions in African countries [12]. Therefore, the case of a 14-year-old girl is presented. The patient was admitted to the psychiatric clinic at JUTH, Ethiopia, with severe unexplained emotional and medical complaints, and severely impaired overall functioning. Recognition and management of this patient’s condition are described in this paper. The purpose of this case report is to help develop confidence for mental health providers in managing patients with similar conditions, and to stimulate further evaluation of the conceptual approach in developing countries such as Ethiopia.

## Case report

### Admission

A 14-year-old girl was consulted for psychiatric assessment at the department of paediatric ward of JUTH. She was assessed for a cluster of symptoms including intractable vomiting, inability to eat and walk, and significant loss of weight with emaciation.

### Pathways taken to access service

Greatly concerned, her father had taken her to the nearby hospital, a health care facility three hundred kilometres from Jimma town. From there, she was referred to Addis Ababa, which is approximately 650 kilometres from her home. She was then taken to various prestigious public and private health care institutions, where she was examined for a number of possible medical complaints by methods including CT scans and MRI examination of the spinal cord. All results were normal. The family incurred huge expenses from these tests, and yet her condition remained unexplained, and she did not receive the necessary help. In desperation, and losing hope, her father took her back to the first hospital, and from there, she was referred to JUTH. At JUTH, she was first contacted and admitted to the paediatric ward, from which she was finally transferred to the psychiatry clinic through a consultation process.

#### Somatic findings

The examining clinician noted sunken eyeballs, generalised body weakness, and significantly decreased overall muscular mass. Slight abdominal tenderness was detected during physical examination and vital signs were within normal range. With support, she was able to sit and stand upright, but was not able to take a single step, or stand for long. At admission, her weight was 22 kg, her height 132 cm, and body mass index 12.6, which was below the 3rd percentile [13]. Consequently, a nasogastric tube was inserted for feeding and treatment for suspected Peptic Ulcer Disease commenced. It was observed that she was able to move her legs to some degree whenever she was distracted. After 2 weeks of thorough investigation, including neurological examination, there were no findings suggestive of any medically explained illnesses, and as she appeared severely mentally disturbed, she was transferred to the psychiatric ward. The referral diagnosis was suspected as early onset schizophrenia, and the differential diagnosis was conversion disorder.

## Admission to psychiatry ward

### History

The girl discussed in this case report is the oldest female child of four children in a Protestant family. She was responsible for the household chores, which is a culturally accepted age-appropriate task in Ethiopia, even for younger girls. Getting to school meant a 2-h walk every day for this fifth grade student.

Her father reported that she had started to complain of low back pain, pain in her head, and a decrease in appetite 4 months prior to developing other clinical features. Moreover, she had difficulty performing household chores. She was complaining of a sensation of “a moving live animal” in her abdomen, which is a culturally-appropriate sensation and belief during illness. Soon after reporting this, she felt too tired to go to school. Family members encouraged her to attend her classes, but this subsequently became impossible. She remained in this condition for 2 months, blaming herself for not being able to continue her education or help her mother. She was sad and cried intermittently, blaming herself for being unable to accomplish what was expected from her and lagging behind her classmates at school—both of which contributed to her feelings of inadequacy. Two months before admission she developed intractable vomiting and frequent retching, which became a great worry for her parents. Soon after this, she developed difficulty in moving her legs, and needed help to move.

#### Mental state finding

The girl was carried to the psychiatry clinic by her father, as she was unable to walk on her own. At the bedside examination, she appeared chronically sick-looking, and was very weak. She displayed severe muscular atrophy of her legs and was retching incessantly. The patient could maintain good eye contact, and understand what others were trying to communicate, including clinicians. She did not appear depressed and was responsive. Her speech was coherent but remarkably low in volume and tone, and monotonous. She was irritable, crying, and appeared to be in significant emotional distress. Behaving agitated and restless, she seemed to be in a state of hyperarousal. Her stage of development was prepubertal.

#### Diagnostic considerations

Differential diagnoses: the overall functioning was severely impaired. Patients being underweight due to malnourishment are seen frequently in clinical settings. However, Anorexia Nervosa is unusual in developing countries. The severe atrophy of the patient’s muscles indicated a longer period of inactivity, and a disease of the muscles was suspected. There was no motor pattern, which indicated a neurological disorder. The gastrointestinal symptoms might have been an indicator of a Peptic Ulcer Disease, which can occur as a stress reaction. Predominant symptoms of delirium, such as impaired consciousness and cognitive abilities, were not present. Based on the father’s report of past symptoms such as loss of energy, loss of appetite, and thoughts of unworthiness, the patient appeared to have been depressed at the initiation of symptoms. Early onset psychosis is a rare condition in adolescents, which is characterised by disturbed behaviour and disturbances in emotions, social contact, and thoughts. Initially, her complaint of having a moving animal in her stomach was considered as an expression of somatic hallucinations and derealisation. An antipsychotic treatment with haloperidol was started [14].

### Working diagnosis

The section “significant emotional or medically unexplained complaints” of the WHO mental health GAP intervention guide summarises best the problems this adolescent presented. People in this category have anxiety-related, depressive, or medically unexplained somatic symptoms [10], and may experience a mental disorder not covered in the WHOmhGAP, e.g. somatoform disorder, generalized anxiety disorder, post-traumatic stress disorder, acute stress reaction, mild depression, etc.

### Management: symptom orientated supportive therapy

At the intake of the patient to psychiatric ward, as well as previously to the paediatric ward, there were great deals of confusion. There was uncertainty as to whether the patient suffered from an organic disease or a mental health disturbance. Discussion continued regarding the appropriate setting for the patient—the paediatric clinic or the psychiatric clinic. Finally, arrangements were made to consult a paediatrician every 2 days, and to admit the patient to the psychiatry ward. The role of the child psychiatrist was to participate in the assessment and to encourage a stepwise approach irrespective of a definite final diagnosis. The main goal in treating this patient was to ensure safety and to improve functioning.

The first step was to contact the patient and to build an alliance with her family. Her father and her uncle were with her during her hospitalisation. They were continuously involved in all procedures, and supported cooperation between the girl and the staff. In addition, the patient’s father gave consent for the team to make successive video recordings for use as teaching material. The children’s nurse sat at the girl’s bedside for short intervals several times a day to offer consolation and to make her feel comfortable. The nurse offered material for drawing to distract the patient from crying and praised her when she participated. The girl’s crying attracted patients and relatives from the ward who wanted to come in and to see what the matter was, and large audiences around her bed reinforced her behaviour in a negative way. Therefore, staffs were advised to remove people from what would often become an overcrowded room. From the video recordings, it was demonstrated that members of the staff tried to calm her down by speaking louder if the girl was crying. This approach was not successful.

In contrast, when the girl was given material for drawing, and the attention of both the nurse and the girl was directed to this task, her voice lowered, and she was able to be calmed down for some minutes. Giving her attention when she was quiet was an effective strategy. She was allowed to indicate when she wanted to stop an activity, even after a short interval, and to have rest. Due to the continuous guidance and constructive feedback the girl was able to build a relationship with the children’s nurse. Communication between them was mainly nonverbal, with some translations from clinicians and father. She became noticeably more confident. During the early stages, the girl’s father supported her in achieving a sitting position in her bed. Later on, she was able to sit by herself in her bed, and subsequently outside the bed. Observation showed that the girl was not vomiting, but crying in despair. To facilitate self-regulation, the children’s nurse demonstrated how to breathe slowly, and the girl was able to take over and to settle down. The girl’s drawings of scenes with flowers, and of a coffee ceremony, were well elaborated. She was able to understand tasks well and showed a good ability to plan. She was given the task of making another drawing on her own for the next day and was able to complete this task.

For sensory stimulation, she was asked to take water in her mouth without swallowing several times a day and to have washed her feet. These tasks were taken over by the father. Nutrition was given by infusion, and after 3 days, loss of weight was stated. A check-up showed that the amount of calories was not sufficient and feeding was continued by nasogastric tube. Mashed soup was given in small portions (1 l in portions of 5 ml every 10 min), initially by the children’s nurse, then by her father. To explore her perception and cognition, she was asked how it felt in her stomach.

Her drawings and some school-related tasks such as doing calculations gave an approximate indication of her age-appropriate cognitive development. From day to day the patients crying diminished and finally stopped whereas at the same time she was able to start taking care of herself. Her thought content was demonstrated to be clear, and her good social and cognitive abilities convinced the clinicians that she had no psychosis. Haloperidol medication was stopped after 4 days. Her attention span became longer and the duration of interactions with her could be extended. For every successful step in her treatment, she was given positive feedback. When the patient was able to perform a certain task, another more demanding next step would follow. After 6 days, she was able to swallow and to drink. She was praised by family members and staff for this progress, and she was very proud herself. After 8 days, she could start to eat solid food, and was therefore offered her favourite food. Within the same week, she was observed to be euthymic, happy, smiling, and engaging in appropriate verbal, as well as non-verbal, communication. She was energetic, greeting the staff by offering her hand, and had started sitting by herself with no support.

Further steps included sitting on a chair and joining a group of patients doing occupational activities. When she was painting in a group situation, the persons sitting next to her tried to guide her on how to paint. She resisted and instead did it in her own way. This was an example of her assertive behaviour, which was recorded on video. Four days prior to leaving for home, she was seen attempting to stand with and without support. The next day, she was able to walk by herself needing no support at all. To the surprise of her father she began walking as she used to do during her pre-morbid state. He expressed his happiness by saying, “I couldn’t believe my eyes and my sacrifice is not in vain”. At this stage in the patient’s recovery, her father took her home, despite medical advice, as he was about to run out of money.

### Mental state at discharge

The improvement in the patient’s overall functioning was dramatic: partial symptom relief was attained; the patient was able to eat normally; social contact was adequate; she showed a good mood and affect; and her cognitive functions were adequate for her age. However, stabilisation and recovery was still necessary: emotional—to strengthen self-confidence; nutrition—to regain normal weight; motor functions—training to walk; and social—to return to school. The protocol of the stepwise approach of interventions is given in the Table 1.

**Table 1:** Protocol of a stepwise approach: problem-oriented interventions: social-emotional, eating, motility

Day	Safety	Social-emotional	Eating	Motility	Autonomy
1	Bedside evaluation mental state antipsychotic medication	Crying, retching	Passive: infusion	Sits in bed if supported	
2	Build alliance with family, establish supportive relationship with patient	Nurse gets in contact with patient, offers contact, occupation at frequent short intervals	Infusion	Continued, able to sit alone in bed	Patient decides when to stop (some minutes)
3	Weight control, check caloric supply nasogastric tube	Breathing exercises by nurse, explore cognition: how does it feel in your stomach? Cognitive stimulation: school work at bedside	Sensory input, water in mouth without swallowing, mashed soup via tube, initially administered by nurse, then by father	Sensory input wash feet twice a day by father	Breathe slowly for self regulation reflection on inner state
4	Weight gain, antipsychotic medication stopped	Occupation continued, variety of tasks	Continued	Sits at bedside for a moment	Crying diminishes
5		Continued variety of tasks, longer attention span		Continued	Crying stops, is getting calm
6	Safe relationship to nurse	Praised by family and professionals	Active: able to swallow liquid		Active intake of food—very proud
7		Takes part in group painting		Sits in group at table	
8			Active: eats solid food nasogastric tube removed	Passive: massage of legs; active: guided movements, relaxation exercises	Expresses wish for favourite food, active movements of legs despite being anxious
9		Occupation for 1 h needle work fast, skilful		Able to sit in group for 1 h, stands with support	Able to ask for material, persists on own ideas in painting, able to say no, assertive behaviour, confident
10				Some steps on her own without support	

#### Follow up

Due to the long distance between the patient’s home and JUTH, it was not possible to follow up on this patient in person. However, after discharge and during 6 months of subsequent contact by phone, (three times for the first month, two times in the second and third consecutive months, and once after 6 months) her father reported that she was in a good state. She was able to continue her education and was helping her mother at home. Her parents were afraid that her working as before might worsen their daughter’s condition, but this could not be checked because of lack of further follow up.

## Discussion

### Recognition

Initially, the condition of the adolescent girl was viewed as life threatening due to the marked loss of weight. A general medical examination and essential investigations had been conducted. However, no organic disease could be found that fully explained the presence of the symptoms. The adolescent did not show signs of moderate or severe depression, or of suicidal thoughts or self-harm. In the patient’s history, no exposure to extreme stressors was mentioned. Overall, the patient’s medical symptoms were unexplained. However, it was obvious that she was in severe distress. According to the WHOmhGAP, people with “significant emotional or mentally unexplained complaints” may have mental disorders not covered in the manual, such as somatoform disorders, generalised anxiety disorder, mild depression or others.

There are different languages and ways of thinking within the medical field between somatic-oriented professions and psychiatrists. Translation processes are therefore necessary, and additional chains of translation processes are necessary to communicate with patients in an adequate way. Observation, especially if supported by video documentation, is a direct approach, which can be shared across cultural views.

Even in a situation of high urgency, it is worthwhile to take time to observe the patient, from a supportive attitude approach. In the event of feeling lost in the evaluation process, and having little idea about the right diagnosis for a particular child, Lempp et al. [15] recommend getting back to the description of the problems. These authors strongly advise never to leave a family with just the diagnostic information but always to present treatment options and convey hope [15]. The young patient presented in this case report, her family, and local staff regained hope and were able to adopt a positive attitude towards the healing process.

In the Diagnostic and Statistical Manual of Mental Disorders, Fifth Edition (DSM 5, 2013) a group of disorders has as a common feature the “prominence of somatic symptoms associated with significant distress and impairment” [16], which are summarised in the chapter “somatic symptom and related disorders”. The motor problems of the patient discussed in the case report were not consistent with medical psychopathology. Therefore, conversion disorder had to be considered. The criteria of conversion disorder are: (A) one or more symptoms of altered voluntary motor or sensory function; (B) clinical findings provide evidence of incompatibility between the symptom and recognised neurological or medical conditions; (C) the symptom or deficit is not better explained by another medical or mental disorder; (D) The symptom or deficit causes clinically significant distress or impairment in social, occupational, or other important areas of functioning or warrants medical evaluation [16].

In the previous version of the classification system DSM-IV, conversion disorder is a subcategory of the broad category “somatoform disorders” [17, 18]. In the International Classification of Diseases (ICD-10) classification of mental and behavioural disorders: clinical descriptions and diagnostic: World Health Organization, these symptoms are classified as “dissociative disorder or conversion disorder” with symptom types “motor symptom—weakness, swallowing symptoms and sensory symptoms” [19]. Conversion disorder is often associated with dissociative symptoms, such as depersonalisation, derealisation, and dissociative amnesia [16].

Conversion disorders are characterised by the partial or complete lack of the normally integrated functions of memory, identity, perception of the environment, and control of physical movements [19, 20]. The onset of these characteristics is known to occur in childhood or adulthood, and is often a result of traumatic life events such as childhood emotional, physical, or sexual abuse, or other adverse life events. In Sub-Saharan countries, one has to keep in mind the possibility of female circumcision in children [21]. A recent survey from Jimma town in Ethiopia showed that there is a positive attitude amongst the public towards female circumcision, despite it being prohibited by the law. From this perspective, the family of a child undergoing female circumcision would not necessarily regard it as a traumatic event [22]. For clinicians, it can be difficult, even impossible, to explore the issue, as doing so risks angering the family. For the young patient presented in the case report, no psychological stressor could be identified. The final diagnosis of the patient was conversion disorder.

The frequency of dissociative experiences peaks during latency years and declines between early adolescence and young adulthood [23]. Many epidemiological studies have shown that the incidence and prevalence of conversion/dissociative disorders in adults vary across countries and communities, and is generally more prevalent in developing countries than western developed communities [24]. In the clinic for child psychiatry in the Ethiopian capital of Addis Ababa, adolescents with dissociative disorder are seen and treated frequently (Baheretibeb, personal communication) [25].

The term “model” implies that particular phenomena can be represented (i.e., modelled) in multiple ways. Thus a DSM model may use different terms and criteria compared to an ICD model, but both may be useful for different purposes and/or in different systems of care [26]. The management of psychiatric disorders is not something that is included as part of the classification systems ICD 10 of the WHO, and the DSM-IV or V of the American Psychiatric Association. Hence, the WHOmhGAP was chosen over ICD and the DSM for training purposes, and for management in the MSc programme.

There are currently only a few case reports that discuss similar conditions in adolescents presented in this case report. Examples of similar reports include a 17-year-old male adolescent from Nigeria suffering from sickle cell anaemia, and who developed psychosis. A possible explanation given for the development of psychosis was brain infarcts, which may have induced this mental disorder [27]. The focus of this report was on the interaction between an organic disease and the mental disorder psychosis. Another example described a 14-year-old French girl who was admitted to a hospital for a rare form of Dissociative Disorder called “Ganser syndrome” [28]. The patient experienced two episodes, the second of which was accompanied by depressive symptoms. The French authors discuss whether—as in their patient—an episode of Dissociative Disorder, must be regarded as a precursor of depression or bipolar disorder. They stress that it can be difficult in adolescents to differentiate between derealisation as a phenomenon of normal development, schizophrenia, depression, and dissociative disorder. Their focus was course and development of dissociative disorder [28]. Finally, a 14-year-old male Kurdish student from Iraq was diagnosed as having Anorexia Nervosa [29]. He was hospitalised and responded well to medical and psychiatric treatment. In this particular case, contrasting cultural influences such as the Arabian Muslim culture on one hand, and a Western influence via television, Internet, and periodicals on the other hand, can be regarded as precipitating factors.

### Management

The core problems of the patient presented in the case report were impairment of functions of memory, identity, perception of the environment, and control of physical movements. The basic principle of treatment was to provide a safe environment and restore autonomy, turning from passive to active.

#### Immediate and short-term psychosocial interventions

The management of the patient’s treatment made use of general principles of care and some elements of “advanced interventions”, such as relaxation training and social skills therapy, as described in the WHO MhGAP intervention guide [10]. Relaxation training involves training a person in techniques such as breathing exercises to elicit the relaxation response. Social skills therapy helps rebuild skills and coping in social situations to reduce distress in everyday life, and uses social tasks, encouragement, and positive social reinforcement to help improve ability in communication and social interactions [10]. Tasks are aimed at being meaningful and oriented to the interests of the person, and the difficulty and the duration of any task have to be adapted to the level of personal capacity to guarantee a successful performance. The challenge is to find the appropriate “dosage” and timing of psychosocial interventions. In terms of medication, after the withdrawal of haloperidol, no other medication was given to the patient [14].

The improvement of the patient in this case report was dramatic. She stayed in the psychiatric ward for 2 weeks, and the majority of the practical work was conducted by the children’s nurse, with assistance from the psychiatrists. Despite gaps in the patient’s history and uncertainty about the diagnosis at the initial stage, the development of the intervention was successful. Reflecting on the girl’s stay in the hospital, it can be noted that her hospitalisation was too short for stabilisation, and thorough examination for any psychological trauma that could have explained her condition. However, after returning to her home, the patient could rely on good parental support.

Similar recommendations for a rehabilitative approach in children are given from authors working in the field of paediatric liaison [17, 18]:

Steps for intervention include;

- A de-emphasis on a final diagnosis,
- Use of benign remedies,
- Reinforcement of wellness [17, 18],
- Reinforcement of well behaviour,
- Encouragement of participation in everyday activities [2].

These authors stress the importance of a close cooperation between all professionals involved, such as school teachers, practitioners, and the patient’s family.

In three women with conversion disorder, physical therapy was part of a rehabilitation program. Movement patterns were corrected using feedback and praise. The therapy program was progressively more difficult and resulted in symptom relief [30]. In child and adolescent conversion disorders, the evidence of physiotherapy is limited due to the lack of systematic studies [31].

#### Long-term management

Persons with conversion disorders can recover within a short time. In the long-term, however, relapses can occur. Therefore, it is preferable to have fixed follow-up appointments spaced apart by long intervals. The concern of the parents about a possible relapse in their child provides a starting point for further counselling, and an overprotective attitude may develop if parents are especially anxious about a relapse. In Ethiopia, severe mental illness is quite often attributed to spiritual factors such as possession, bewitchment, or the evil eye [6, 32]. Extending the case history during follow-up visit helps in the understanding of predisposing, precipitating, and protecting factors. The health professional has to ask, therefore, about the patient’s (and family’s) health beliefs. In the case of a reappearance of symptoms, further treatment can be offered.

#### Capacity building

Coming into a clinical setting from outside it can take time to be able to appreciate and understand the cultural aspects of another environment. For example, in Ethiopia relatives are expected to care for the patient by themselves, while nurses are responsible for distributing medication. The guest lecturers at JUTH had to find their place in the setting of the psychiatric clinic after they first arrived. Both of them, the child psychiatrist and children’s nurse, had to negotiate with clinicians and staff on the one hand and the family on the other hand to explain their ideas how to proceed. The role of the children’s nurse was unusual for the local nurses. She offered a relationship by sitting at the bedside of the patient without being anxious herself. Both guests observed what was going on with the patient and guided the perception of the staff. Both stressed the successes of even small interventions, and after initial improvement, the approach was accepted.

While the clinicians had considerable experience in drug treatment, there was inadequate time for practice, and little experience amongst them for psychosocial interventions for children and adolescents. The treatment approach to the health problems of this young patient was developed in successive discussions between the local clinicians, and the guest lecturers, and turned out to be successful. These two groups combined their knowledge of the cultural background, the local situation, and their experience and intuition in dealing with adolescents. During the course of treatment, the video recordings served to allow for observation, assessment, and management of the patient. In the work-up, key elements of the intervention could be identified by evaluation of the video recordings and shown for teaching purposes. The participants in the programme gave feedback, and stated that they felt no longer anxious but confident in treating child patients.

Confidence as a health care practitioner develops from training, and one’s own experience of successful interventions. To evaluate an intervention’s effectiveness, systematic follow-up is needed. Until now, only a small number of studies have attempted to evaluate the individual service processes necessary for successful implementation of community mental health care [33]. To test for generalisation, the intervention would have to be applied to other patients with a similar condition.

## Conclusion

The primary message of this case report is that significant emotional and medically unexplained complaints can be treated successfully with psychosocial interventions in a resource-limited setting. However, gaps in the information-gathering process have to be accepted at this early stage. The aim of the publication of this case report is to make mental health services for children accessible and visible. The implementation of child mental health services in a region where there have previously been no child mental health professionals is a complex and challenging process. Case-based learning relying on local expertise is generally the most suitable approach in meeting the local needs, and for developing mental health services for children and adolescents. The development of specialised care should occur prior to, or in parallel with, the delivery of mental health services by primary care.

### Consent

During the inpatient stay, the father of the patient gave consent for the team to take video recordings to use as a teaching material. The father had also consented for the publication of this case report, provided the information was anonymous and kept confidential.

## Appendix: List: Essential treatment elements (WHOmhGAP intervention guide 2010, [10])

### Ensure safety

Ensure basic physical needs are met:

Nutrition: guarantee survival by sufficient caloric intake.

- Build up eating behaviour.

In acute distress offer basic psychological support:

- Listen without pressing the person to talk.
- Assess needs and concerns.
- Protect from further harm.

### Restore functionality: problem oriented approach

Social-emotional:

- Give comfort and support self-regulation by relaxation exercises.
- Activate behaviour.
- Encourage gradual return to normal activities.
- Provide or mobilize social support.

Restore motility: Encourage passive and active movements by structured physical activity.
